# Supplementary material for: Emerging Highly Pathogenic Avian Influenza H5N1 Clade 2.3.4.4b Causes Neurological Disease and Mortality in Scavenging Ducks in Bangladesh
Source: Vet Sci. 2025 Jul 23;12(8):689. doi: 10.3390/vetsci12080689 (PMC12389831; doi:10.3390/vetsci12080689)
Supplement: Supplementary file 1 [file vetsci-12-00689-s001.zip › Supplemental Figure S1.pdf]

Supplemental Figure S1

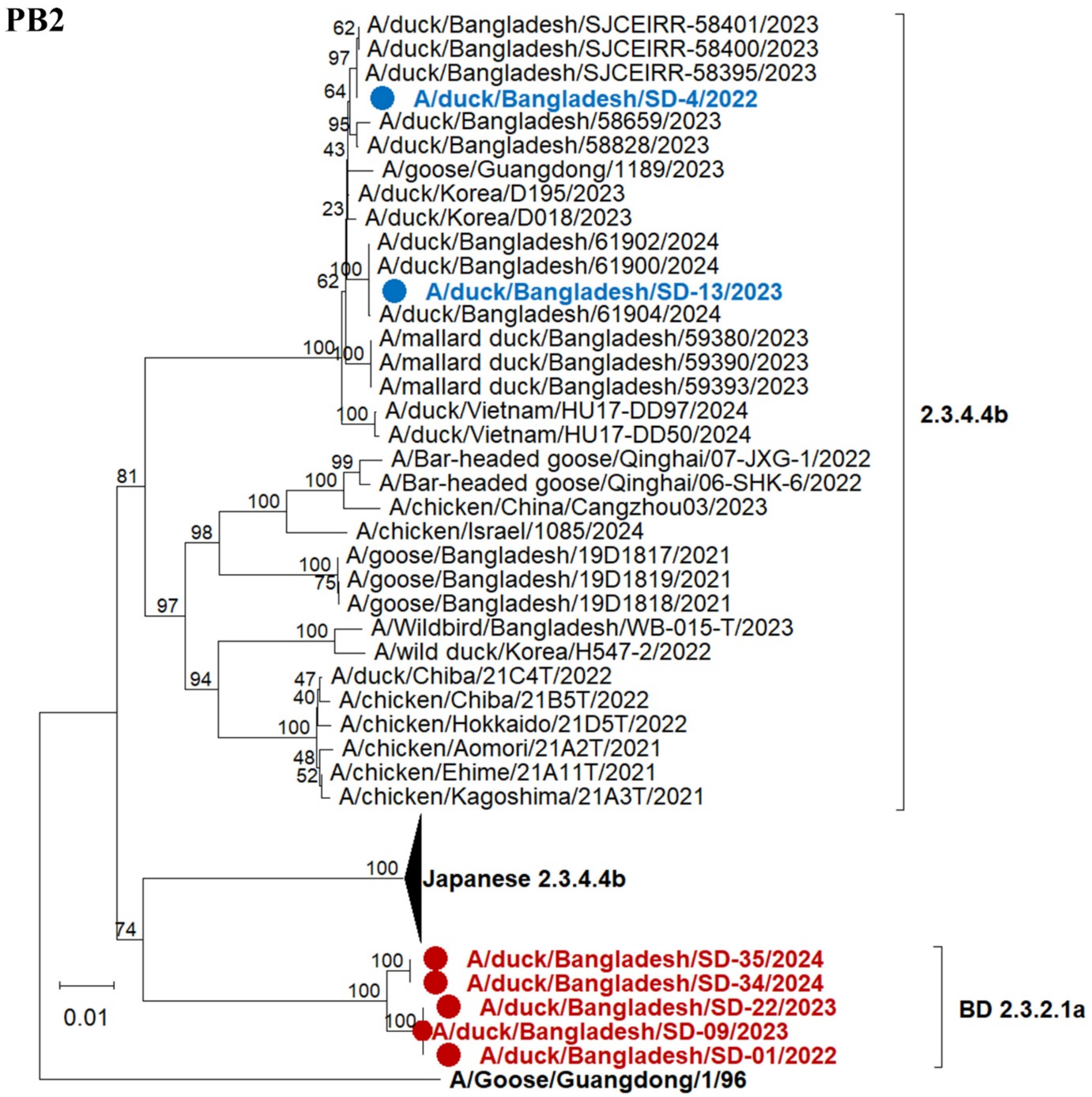

PB1

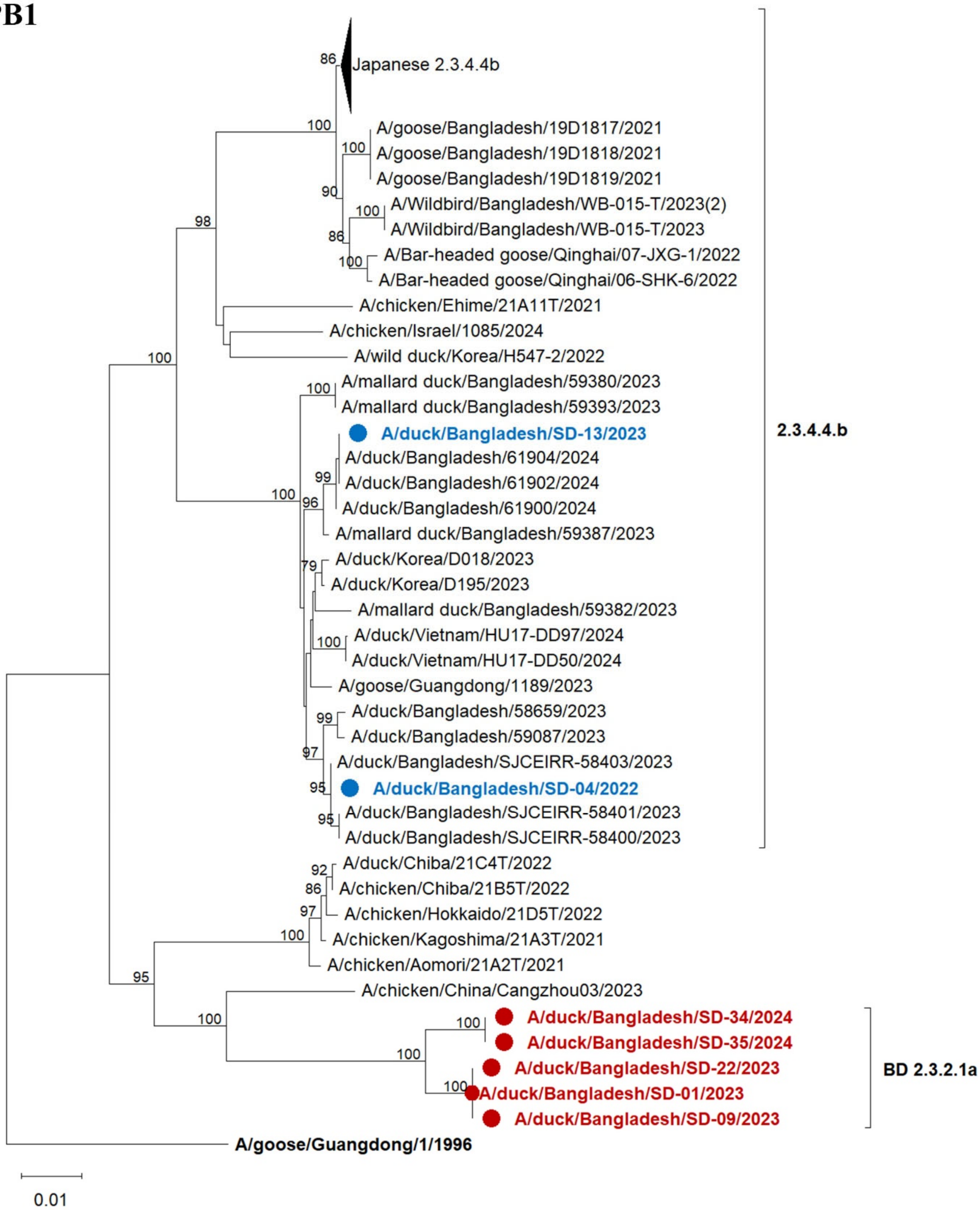

Japanese 2.3.4.4b
A/goose/Bangladesh/19D1817/2021
A/goose/Bangladesh/19D1818/2021
A/goose/Bangladesh/19D1819/2021
A/Wildbird/Bangladesh/WB-015-T/2023(2)
A/Wildbird/Bangladesh/WB-015-T/2023
A/Bar-headed goose/Qinghai/07-JXG-1/2022
A/Bar-headed goose/Qinghai/06-SHK-6/2022
A/chicken/Ehime/21A11T/2021
A/chicken/Israel/1085/2024
A/wild duck/Korea/H547-2/2022
A/mallard duck/Bangladesh/59380/2023
A/mallard duck/Bangladesh/59393/2023
A/duck/Bangladesh/SD-13/2023
A/duck/Bangladesh/61904/2024
A/duck/Bangladesh/61902/2024
A/duck/Bangladesh/61900/2024
A/mallard duck/Bangladesh/59387/2023
A/duck/Korea/D018/2023
A/duck/Korea/D195/2023
A/mallard duck/Bangladesh/59382/2023
A/duck/Vietnam/HU17-DD97/2024
A/duck/Vietnam/HU17-DD50/2024
A/goose/Guangdong/1189/2023
A/duck/Bangladesh/58659/2023
A/duck/Bangladesh/59087/2023
A/duck/Bangladesh/SJCEIRR-58403/2023
A/duck/Bangladesh/SD-04/2022
A/duck/Bangladesh/SJCEIRR-58401/2023
A/duck/Bangladesh/SJCEIRR-58400/2023
2.3.4.4.b
A/duck/Chiba/21C4T/2022
A/chicken/Chiba/21B5T/2022
A/chicken/Hokkaido/21D5T/2022
A/chicken/Kagoshima/21A3T/2021
A/chicken/Aomori/21A2T/2021
A/chicken/China/Cangzhou03/2023
A/duck/Bangladesh/SD-34/2024
A/duck/Bangladesh/SD-35/2024
A/duck/Bangladesh/SD-22/2023
A/duck/Bangladesh/SD-01/2023
A/duck/Bangladesh/SD-09/2023
BD 2.3.2.1a
A/goose/Guangdong/1/1996
86
100
100
90
100
86
100
98
100
100
100
99
96
79
100
99
97
95
95
92
86
97
100
95
100
100
100
100
0.01

PA

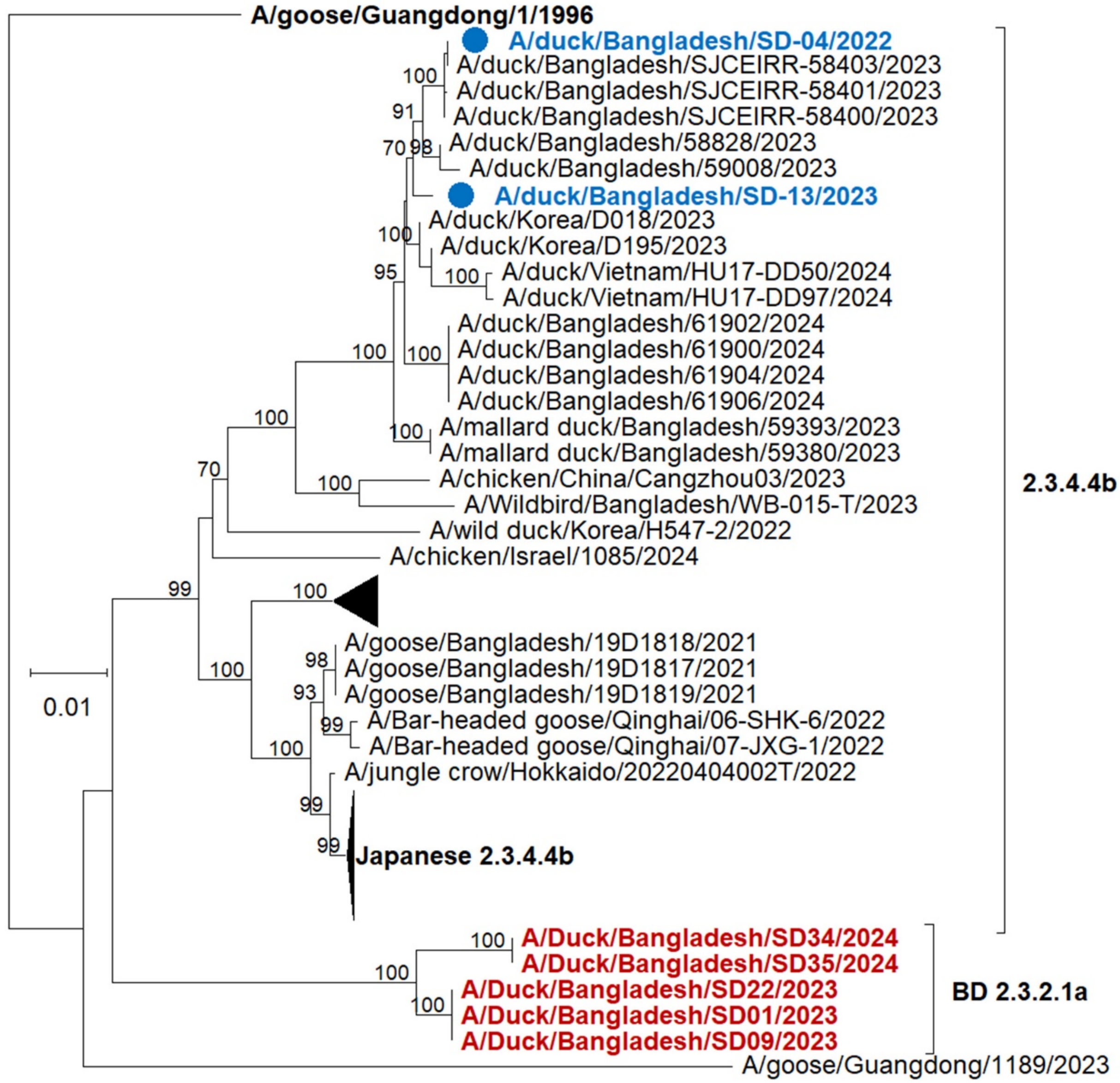
A/goose/Guangdong/1/1996
A/duck/Bangladesh/SD-04/2022
A/duck/Bangladesh/SJCEIRR-58403/2023
A/duck/Bangladesh/SJCEIRR-58401/2023
A/duck/Bangladesh/SJCEIRR-58400/2023
A/duck/Bangladesh/58828/2023
A/duck/Bangladesh/59008/2023
A/duck/Bangladesh/SD-13/2023
A/duck/Korea/D018/2023
A/duck/Korea/D195/2023
A/duck/Vietnam/HU17-DD50/2024
A/duck/Vietnam/HU17-DD97/2024
A/duck/Bangladesh/61902/2024
A/duck/Bangladesh/61900/2024
A/duck/Bangladesh/61904/2024
A/duck/Bangladesh/61906/2024
A/mallard duck/Bangladesh/59393/2023
A/mallard duck/Bangladesh/59380/2023
A/chicken/China/Cangzhou03/2023
A/Wildbird/Bangladesh/WB-015-T/2023
A/wild duck/Korea/H547-2/2022
A/chicken/Israel/1085/2024
A/goose/Bangladesh/19D1818/2021
A/goose/Bangladesh/19D1817/2021
A/goose/Bangladesh/19D1819/2021
A/Bar-headed goose/Qinghai/06-SHK-6/2022
A/Bar-headed goose/Qinghai/07-JXG-1/2022
A/jungle crow/Hokkaido/20220404002T/2022
Japanese 2.3.4.4b
2.3.4.4b
A/Duck/Bangladesh/SD34/2024
A/Duck/Bangladesh/SD35/2024
A/Duck/Bangladesh/SD22/2023
A/Duck/Bangladesh/SD01/2023
A/Duck/Bangladesh/SD09/2023
BD 2.3.2.1a
A/goose/Guangdong/1189/2023
0.01
100
91
70
98
100
95
100
100
100
100
100
70
99
100
100
98
93
99
100
99
99
100
100
100

NP

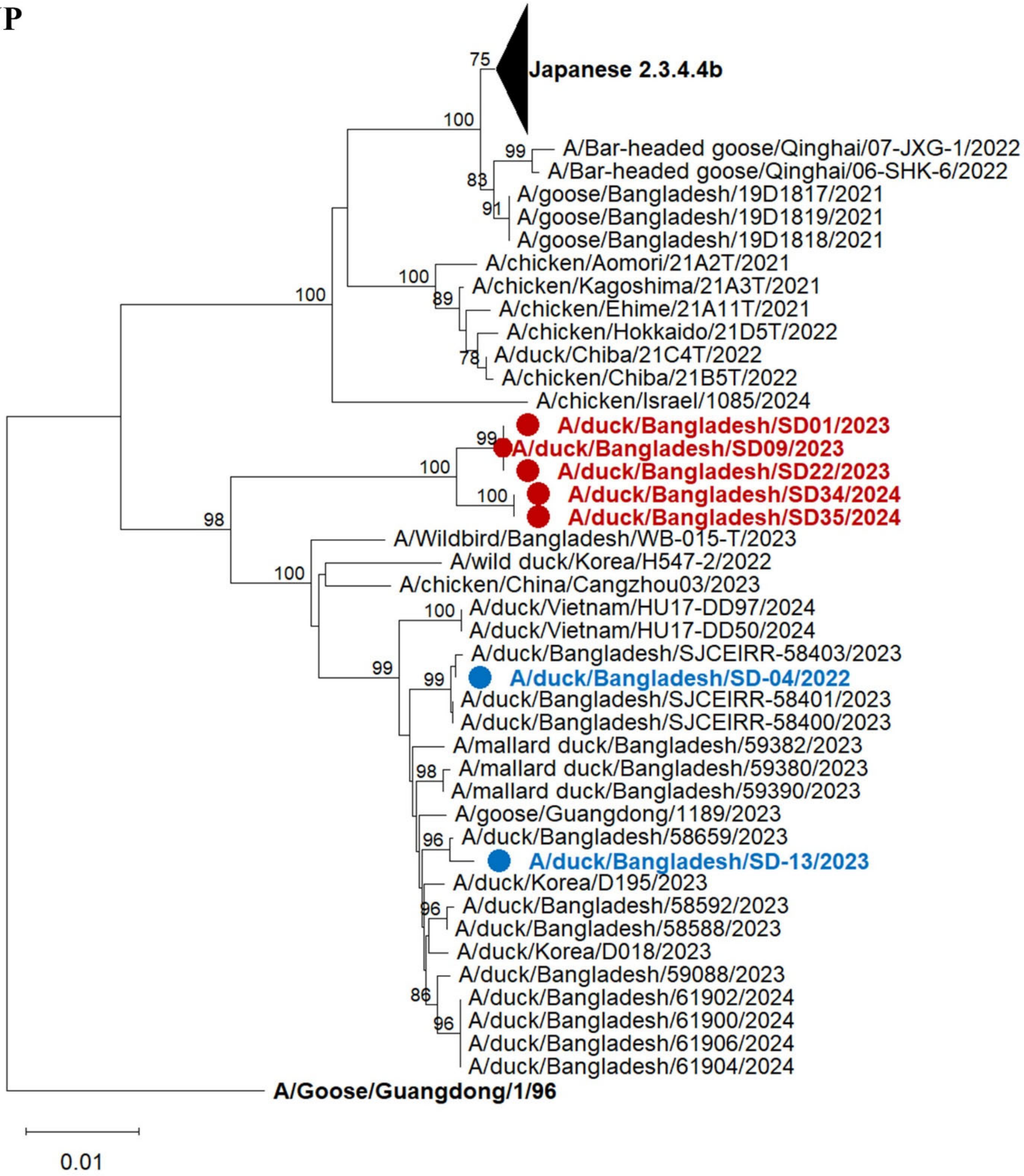

Japanese 2.3.4.4b
A/Bar-headed goose/Qinghai/07-JXG-1/2022
A/Bar-headed goose/Qinghai/06-SHK-6/2022
A/goose/Bangladesh/19D1817/2021
A/goose/Bangladesh/19D1819/2021
A/goose/Bangladesh/19D1818/2021
A/chicken/Aomori/21A2T/2021
A/chicken/Kagoshima/21A3T/2021
A/chicken/Ehime/21A11T/2021
A/chicken/Hokkaido/21D5T/2022
A/duck/Chiba/21C4T/2022
A/chicken/Chiba/21B5T/2022
A/chicken/Israel/1085/2024
A/duck/Bangladesh/SD01/2023
A/duck/Bangladesh/SD09/2023
A/duck/Bangladesh/SD22/2023
A/duck/Bangladesh/SD34/2024
A/duck/Bangladesh/SD35/2024
A/Wildbird/Bangladesh/WB-015-T/2023
A/wild duck/Korea/H547-2/2022
A/chicken/China/Cangzhou03/2023
A/duck/Vietnam/HU17-DD97/2024
A/duck/Vietnam/HU17-DD50/2024
A/duck/Bangladesh/SJCEIRR-58403/2023
A/duck/Bangladesh/SD-04/2022
A/duck/Bangladesh/SJCEIRR-58401/2023
A/duck/Bangladesh/SJCEIRR-58400/2023
A/mallard duck/Bangladesh/59382/2023
A/mallard duck/Bangladesh/59380/2023
A/mallard duck/Bangladesh/59390/2023
A/goose/Guangdong/1189/2023
A/duck/Bangladesh/58659/2023
A/duck/Bangladesh/SD-13/2023
A/duck/Korea/D195/2023
A/duck/Bangladesh/58592/2023
A/duck/Bangladesh/58588/2023
A/duck/Korea/D018/2023
A/duck/Bangladesh/59088/2023
A/duck/Bangladesh/61902/2024
A/duck/Bangladesh/61900/2024
A/duck/Bangladesh/61906/2024
A/duck/Bangladesh/61904/2024
A/Goose/Guangdong/1/96
0.01

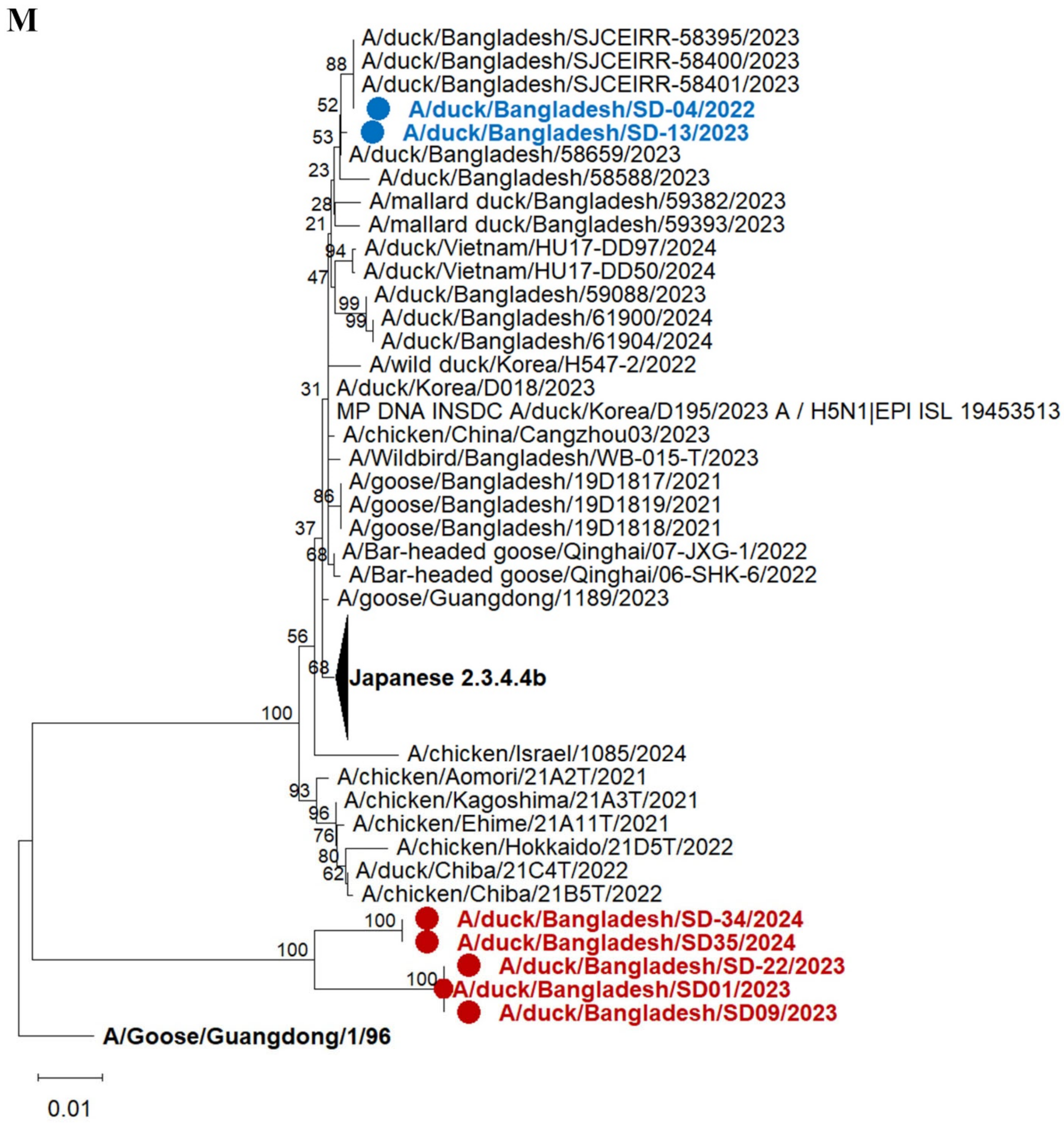
M
A/duck/Bangladesh/SJCEIRR-58395/2023
A/duck/Bangladesh/SJCEIRR-58400/2023
A/duck/Bangladesh/SJCEIRR-58401/2023
A/duck/Bangladesh/SD-04/2022
A/duck/Bangladesh/SD-13/2023
A/duck/Bangladesh/58659/2023
A/duck/Bangladesh/58588/2023
A/mallard duck/Bangladesh/59382/2023
A/mallard duck/Bangladesh/59393/2023
A/duck/Vietnam/HU17-DD97/2024
A/duck/Vietnam/HU17-DD50/2024
A/duck/Bangladesh/59088/2023
A/duck/Bangladesh/61900/2024
A/duck/Bangladesh/61904/2024
A/wild duck/Korea/H547-2/2022
A/duck/Korea/D018/2023
MP DNA INSDC A/duck/Korea/D195/2023 A / H5N1|EPI ISL 19453513
A/chicken/China/Cangzhou03/2023
A/Wildbird/Bangladesh/WB-015-T/2023
A/goose/Bangladesh/19D1817/2021
A/goose/Bangladesh/19D1819/2021
A/goose/Bangladesh/19D1818/2021
A/Bar-headed goose/Qinghai/07-JXG-1/2022
A/Bar-headed goose/Qinghai/06-SHK-6/2022
A/goose/Guangdong/1189/2023
Japanese 2.3.4.4b
A/chicken/Israel/1085/2024
A/chicken/Aomori/21A2T/2021
A/chicken/Kagoshima/21A3T/2021
A/chicken/Ehime/21A11T/2021
A/chicken/Hokkaido/21D5T/2022
A/duck/Chiba/21C4T/2022
A/chicken/Chiba/21B5T/2022
A/duck/Bangladesh/SD-34/2024
A/duck/Bangladesh/SD35/2024
A/duck/Bangladesh/SD-22/2023
A/duck/Bangladesh/SD01/2023
A/duck/Bangladesh/SD09/2023
A/Goose/Guangdong/1/96
88
52
53
23
28
21
94
47
99
99
31
86
37
68
56
68
100
93
96
76
80
62
100
100
100
0.01

NS

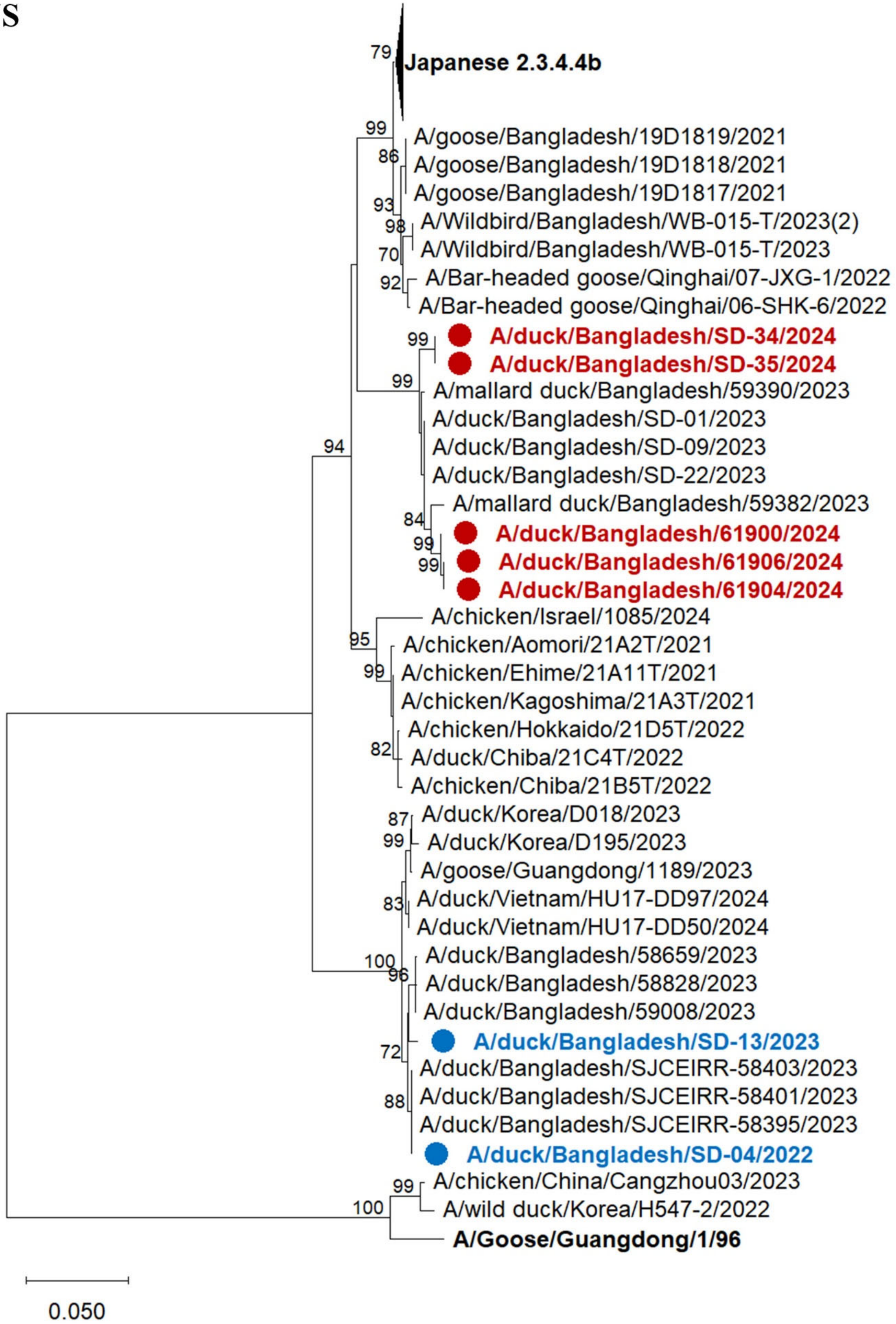

Japanese 2.3.4.4b
A/goose/Bangladesh/19D1819/2021
A/goose/Bangladesh/19D1818/2021
A/goose/Bangladesh/19D1817/2021
A/Wildbird/Bangladesh/WB-015-T/2023(2)
A/Wildbird/Bangladesh/WB-015-T/2023
A/Bar-headed goose/Qinghai/07-JXG-1/2022
A/Bar-headed goose/Qinghai/06-SHK-6/2022
A/duck/Bangladesh/SD-34/2024
A/duck/Bangladesh/SD-35/2024
A/mallard duck/Bangladesh/59390/2023
A/duck/Bangladesh/SD-01/2023
A/duck/Bangladesh/SD-09/2023
A/duck/Bangladesh/SD-22/2023
A/mallard duck/Bangladesh/59382/2023
A/duck/Bangladesh/61900/2024
A/duck/Bangladesh/61906/2024
A/duck/Bangladesh/61904/2024
A/chicken/Israel/1085/2024
A/chicken/Aomori/21A2T/2021
A/chicken/Ehime/21A11T/2021
A/chicken/Kagoshima/21A3T/2021
A/chicken/Hokkaido/21D5T/2022
A/duck/Chiba/21C4T/2022
A/chicken/Chiba/21B5T/2022
A/duck/Korea/D018/2023
A/duck/Korea/D195/2023
A/goose/Guangdong/1189/2023
A/duck/Vietnam/HU17-DD97/2024
A/duck/Vietnam/HU17-DD50/2024
A/duck/Bangladesh/58659/2023
A/duck/Bangladesh/58828/2023
A/duck/Bangladesh/59008/2023
A/duck/Bangladesh/SD-13/2023
A/duck/Bangladesh/SJCEIRR-58403/2023
A/duck/Bangladesh/SJCEIRR-58401/2023
A/duck/Bangladesh/SJCEIRR-58395/2023
A/duck/Bangladesh/SD-04/2022
A/chicken/China/Cangzhou03/2023
A/wild duck/Korea/H547-2/2022
A/Goose/Guangdong/1/96
0.050

Supplemental Figure S1: Maximum likelihood phylogenetic tree based on complete six internal gene sequences of contemporary and representative strains. The evolutionary history was inferred utilizing the maximum likelihood method based on the general time-reversible model with 1000 bootstrap replicates and visualized using MEGA XII software. The red color indicates recently characterized 5 strains of clade 2.3.2.1a, while blue color indicates the 2 strains of clade 2.3.4.4b found in Bangladesh. Some other strains of the similar clusters within the branch collapsed.
