# Supplementary material for: Emerging Highly Pathogenic Avian Influenza H5N1 Clade 2.3.4.4b Causes Neurological Disease and Mortality in Scavenging Ducks in Bangladesh
Source: Vet Sci. 2025 Jul 23;12(8):689. doi: 10.3390/vetsci12080689 (PMC12389831; doi:10.3390/vetsci12080689)
Supplement: Supplementary file 1 [file vetsci-12-00689-s001.zip › Supplement Table 1.pdf]

Supplement Table 1: Accession number of submitted sequence of duck samples to the publicly accessed platform Global Initiative on Sharing All Influenza Data (GISAID).

Strain name	Subtype and clade	Gene	Accession Number (GISAID)
SD-1	H5N1 c2.3.2.1a	PB2	EPI4488097
		PB1	EPI4488098
		PA	EPI4488099
		HA	EPI4488100
		NP	EPI4488101
		NA	EPI4488102
		M	EPI4488103
		NS	EPI4488104
SD-4	H5N1 c2.3.4.4b	PB2	EPI4488105
		PB1	EPI4488106
		PA	EPI4488107
		HA	EPI4488108
		NP	EPI4488109
		NA	EPI4488110
		M	EPI4488111
		NS	EPI4488112
SD-9	H5N1 c2.3.2.1a	PB2	EPI4488113
		PB1	EPI4488114
		PA	EPI4488115
		HA	EPI4488117
		NP	EPI4488116
		NA	EPI4488118
		M	EPI4488119
		NS	EPI4488120
SD-13	H5N1 c2.3.4.4b	PB2	EPI4488121
		PB1	EPI4488122
		PA	EPI4488123
		HA	EPI4488124
		NP	EPI4488125
		NA	EPI4488126
		M	EPI4488127
		NS	EPI4488128
SD-22	H5N1 c2.3.2.1a	PB2	EPI4488129
		PB1	EPI4488130
		PA	EPI4488131
		HA	EPI4488132
		NP	EPI4488133

		NA	EPI4488134
		M	EPI4488135
		NS	EPI4488136
SD34	H5N1 c2.3.2.1a	PB2	EPI4488137
		PB1	EPI4488138
		PA	EPI4488139
		HA	EPI4488140
		NP	EPI4488141
		NA	EPI4488142
		M	EPI4488143
		NS	EPI4488144
SD35	H5N1 c2.3.2.1a	HA	EPI4488145
		NA	EPI4488146
		PB2	EPI4488147
		PB1	EPI4488148
		PA	EPI4488149
		NP	EPI4488150
		M	EPI4488151
		NS	EPI4488152
